# Food Emulsion Gels from Plant-Based Ingredients: Formulation, Processing, and Potential Applications

**DOI:** 10.3390/gels9050366

**Published:** 2023-04-26

**Authors:** Canice Chun-Yin Yiu, Sophie Wenfei Liang, Kinza Mukhtar, Woojeong Kim, Yong Wang, Cordelia Selomulya

**Affiliations:** 1 School of Chemical Engineering, UNSW Sydney, Kensington, NSW 2052, Australia; chun_yin.yiu@student.unsw.edu.au (C.C.-Y.Y.); woojeong.kim@unsw.edu.au (W.K.); cordelia.selomulya@unsw.edu.au (C.S.); 2 Agrotechnology and Food Sciences Group, Wageningen University & Research, Droevendaalsesteeg 4, 6708 PB Wageningen, The Netherlands; sophie.liang@wur.nl; 3 National Institute of Food Science and Technology, University of Agriculture, Faisalabad 38000, Pakistan; kinzaulemaan786@gmail.com

**Keywords:** plant-based, emulsion gel, food application

## Abstract

Recent advances in the understanding of formulations and processing techniques have allowed for greater freedom in plant-based emulsion gel design to better recreate conventional animal-based foods. The roles of plant-based proteins, polysaccharides, and lipids in the formulation of emulsion gels and relevant processing techniques such as high-pressure homogenization (HPH), ultrasound (UH), and microfluidization (MF), were discussed in correlation with the effects of varying HPH, UH, and MF processing parameters on emulsion gel properties. The characterization methods for plant-based emulsion gels to quantify their rheological, thermal, and textural properties, as well as gel microstructure, were presented with a focus on how they can be applied for food purposes. Finally, the potential applications of plant-based emulsion gels, such as dairy and meat alternatives, condiments, baked goods, and functional foods, were discussed with a focus on sensory properties and consumer acceptance. This study found that the implementation of plant-based emulsion gel in food is promising to date despite persisting challenges. This review will provide valuable insights for researchers and industry professionals looking to understand and utilize plant-based food emulsion gels.

## 1. Introduction

Food emulsion gels are ubiquitous in the food industry to create texture or a sensory experience for low-fat products or as a vehicle to deliver functional food ingredients via encapsulation [1,2]. Emulsion gels are semi-solid systems with a gel network structure, often embedded with oil droplets [3]. They integrate the dual characteristics of emulsion and gel, improving both the stability of the emulsion system and the rheological and nutritional properties of a hydrogel, making them a unique and versatile format for developing new foods [4]. The difference between an emulsion gel and a simple emulsion with oil droplets as the inner phase lies in the presence of the gel network. In an ordinary emulsion, oil droplets are dispersed in a continuous phase with emulsifiers to lower the interfacial tension between the two immiscible liquids. However, in an emulsion gel, the dispersed oil droplets are not only stabilized by emulsifiers but are also trapped within a continuous gel network, which provides additional stability and unique characteristics to the system [5].

Emulsion gel systems were originally applied to reduce the fat content of food products by incorporating the gelled water phase while still retaining sensory properties [6]. Emulsion gels can be used to replace fats in a variety of food products, including baked goods, processed meat, dairy products, functional foods, and edible 3D printing inks [7]. They are composed of an oil phase and gel matrix, and the gel matrix comprises components with gel properties, mainly edible proteins and polysaccharides. The generation of emulsion gels is based on the interaction forces between the filler and the matrix, while those without interaction forces require external processing or the addition of emulsifiers [8].

In addition to their potential as a fat replacer, emulsion gels show promising prospects as a delivery medium for functional ingredients through encapsulation [9]. They can effectively inhibit the release of the ingredients and improve the efficiency of bioactive substances, while also controlling the release rate of the encapsulated ingredients. According to research, the use of a protein–polysaccharide for encapsulation may yield better results than systems that utilize only protein or polysaccharides [9]. This hybrid matrix has demonstrated improved loading rates and enhanced stability of encapsulated bioactive molecules. These findings have significant implications for the development of functional foods [10].

Traditionally, emulsion gels are stabilized by animal-sourced ingredients, particularly dairy and meat proteins [10]. However, increasing the global consumption of plant proteins has been widely recognized as a critical approach to ensuring food security and sustainability through 2050 [11]. Despite their potential as a healthy alternative to traditional fats, plant-based emulsion gels have yet to be utilized to their full potential. One reason for this is the poor functionality of plant proteins, particularly in terms of solubility and emulsification capability [12], which is especially true for pea protein [13]. Many recent attempts to produce emulsion gels utilizing plant-based ingredients have proven the possibility to partially or totally replace animal ingredients [14]. Nonetheless, further studies are needed to expand the food applications of plant ingredients [2,15,16]. The properties of emulsion gels are closely related to the dispersed phase and continuous phase, and the content, ratio, and type of both directly impact the final properties of the gel. Therefore, systematic understandings of emulsion formation are still needed to promote the application of plant-based ingredients in emulsion gels.

This paper aims to review the current research on emulsion gels using plant-based ingredients, including the formulation, processing, and potential applications. Specifically, it will focus on the composition and properties of emulsion gels, the interaction forces between the oil phase and the gel matrix, the different processing methods for protein and polysaccharide matrices, and the characterization techniques to analyze food emulsion gels. This review will provide a comprehensive understanding of the current state of knowledge on emulsion gels as well as insights into their potential applications in the food industry.

## 2. Formulation of Plant-Based Food Emulsion Gels

**Figure 1:**
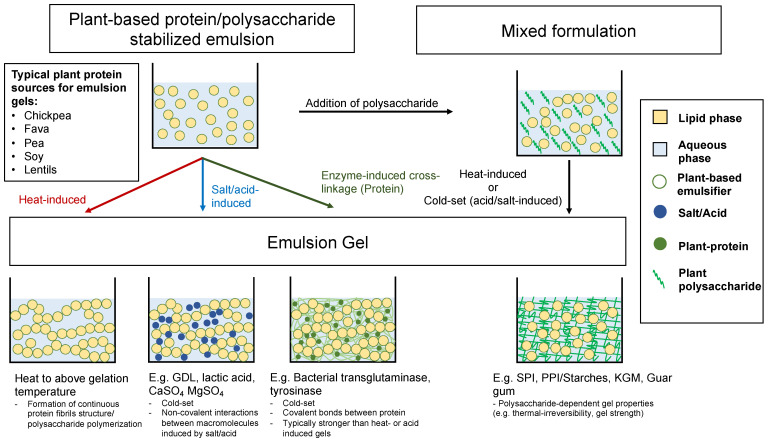
Summary of various methods outlined in Section 2, in which plant-based proteins, polysaccharides, and lipids are used in the formation of plant-based emulsion gel. GDL: Glucono-δ-lactone. SPI: soy protein isolate. PPI: pea protein isolate. KGM: Konjac glucomannan.

The composition of an emulsion gel is fundamental to the stability and physical and mechanical characteristics of the product. Emulsion gels are typically differentiated by the material(s) from which they derive their structure. Emulsion gels may be formed by both proteins and polysaccharides. These hydrocolloids may be used in isolation or in conjunction to stabilize oil-in-water emulsions and provide structure. For emulsion gels that utilized both protein and polysaccharides in their formulation, they may be referred to as “mixed gels” [5,10]. The following sections discuss the functionality of these hydrocolloids and examine their roles in the formation of an emulsion gel and the characteristics they provide to these gels both alone and in a mixed gel formulation. A summary of major techniques for emulsion gel formation and mechanism is presented in Figure 1.

### 2.1. Protein

Protein in a plant-based emulsion gel may be used alone as a structural component or with other components as an emulsifier to stabilize the lipophilic phase [5]. Proteins from legumes and cereals were frequently chosen to make emulsion gels due to their desirable characteristics (emulsion stabilizing and gelation properties) and economy from high-protein plant sources [17]. As such, proteins from soy, peas, chickpeas, fava beans, potatoes, and wheat were among the most studied as structural components and emulsifiers. Additional considerations may be applied when selecting plant proteins, as protein extracts from various sources contain varying protein profiles and allergenicity that may be critical to human health [18]. A prime example of which would be potato protein, where essential amino acid content is among the best in plant-based protein sources [18].

#### 2.1.1. Plant Protein-Based Emulsion Gels

Plant protein-only emulsion gels may be categorized into two major types, characterized by gelation methods. This is namely heat-induced gelation and cold-set gelation with the aid of added coagulants. The formation of plant-protein-based emulsion gels typically follows a two-step process [15]. The first step involves the homogenization of oil and a suspended protein phase to form an emulsion stock. Subsequently, the emulsion is gelled by heat or the addition of coagulants [15,19]. Heat-induced gelation, where globular proteins from plants denature and aggregate to form strong fibrils at elevated temperatures, has been a widely studied method to form a strong, solid-like gel [20]. In heat-set gels, temperatures above 80 °C are typically used for plant protein gelation. Nevertheless, temperatures as high as 95 °C were frequently reported [21,22]. In heat-induced gels, gelation onset temperature may be determined by the oil volume fraction of a gel matrix. In a study on soy protein emulsion gel, a higher oil volume fraction resulted in a lower gelation onset temperature [23]. Structures of heat-induced gels were also found to be more homogenous than those of acid or enzyme-induced gels due to fewer aggregates forming [24]. Moreover, characteristics of heat-induced gels may be further tuned by the total protein content, pH, ionic strength, and ratios between structural proteins within the gel matrix (e.g., glycinin and conglycinin in soy protein gel) [24,25]. However, with the high temperature during heat-induced gelation, heat-set gels may not be suitable if heat-sensitive material is involved.

In response, cold-set gels were investigated to reduce the degradation of heat-sensitive components during gelation. In these gels, gelation was induced by adding coagulants (e.g., acids, salts, and enzymes) to negate heating after the loading of lipids. However, denaturation of plant proteins was still required before cold-setting to expose the sites for aggregation and agglomeration [26]. Acids (e.g., glucono-δ-lactone (GDL) and lactic acid) and salts (magnesium, calcium, and sodium salts) were frequently used to coagulate protein gels. Klost and Drusch [26] reported that lactic acid fermentation of pea protein was able to create a gel suitable for plant-based yogurt [26]. In acid-induced gels, GDL has been a common ingredient in the traditional manufacturing of soft tofu. In emulsion gels, GDL-coagulated soy protein gels were found to exhibit weak rheological behaviors due to non-covalent intermolecular forces that exist within the gel [27]. In salt-induced gels, salt type and salt content are equally significant in gel stability and rheology. Various magnesium and calcium salts (MgCl_2_, MgSO_4,_ and CaSO_4_) were also tested on soy protein isolate by Wang et al. [28]. In their study, magnesium salts (MgCl_2_ and MgSO_4_) showed higher aggregation power than CaSO_4_ as a stronger gel in terms of firmness and rigidity was formed [28]. Optimization of salt content would also provide freeze-thaw stability and rigidity to salt-induced protein gels [14]. Excessive salt was found to promote protein aggregation, leading to a coarse and inhomogeneous gel in NaCl-induced soy protein gels [14].

Enzymes from bacterial sources such as transglutaminase and tyrosinase were used to cross-link proteins from soy, pea, zein, and potato origins. Glusac et al. [29] investigated the use of bacterial tyrosinase to cross-link zein and potato protein in encapsulating olive oil. Additionally, a protease inhibitor in potato protein was added to provide a cross-linkage site with α−zeins resulting in a gel that exhibits superior storage stability to zein-only emulsions after a month of storage at room temperature [29]. In experiments where only the coagulant was altered, enzyme-induced gelation was reported to have a significantly stronger gel structure and elasticity than acid- or salt-induced soy protein emulsion gels. This is indicated by a 31–33% increase in yield stress when comparing the GDL and CaCO_3_ gels to the transglutaminase gel [27]. Enzymes promote the formation of permanent covalent bonds between macromolecules, which results in the creation of a “classical polymer gel” [5]. This is distinct from physical gels (e.g., heat-induced gel), where gel-like properties arise from intermolecular interactions. This fundamental difference explains the typical higher resistance observed for cross-linked gels [5].

#### 2.1.2. Plant Protein as Emulsifier

Dispersion of the oil phase in the form of an emulsion is essential for producing both protein-only and mixed emulsion gels. Proteins could act as a surface-active material that stabilizes the oil/water interface and thereby adsorbs at the interface of the dispersed oil phase, owing to their amphiphilicity from containing both polar and non-polar amino acids [18]. This phenomenon is seen in native isolates of plant proteins [18,30]. Moreover, thermal denaturation of protein in emulsion systems was linked to increasing droplet flocculation due to increased exposure to hydrophobic sites on the protein surface [31]. However, some studies have demonstrated that the pre-denaturation of plant proteins may increase the creaming stability of plant protein-stabilized emulsions [32,33]. This was attributed to the more viscous heat-treated emulsion and the formation of supramolecular structure at the droplet interface, which impeded creaming [32,33]. The emulsification of oil using plant proteins may create an active-filler gel where the dispersed droplets are mechanically linked to the surrounding structural matrix. This is particularly true for heat-set protein gels, as a higher gel strength is typically observed as a result [5].

Since an emulsion is first produced before gelation, it is also important to maximize emulsion stability to give it flexibility for gelation conditions and further processing. The use of dairy proteins to stabilize a hydrophobic phase has been widely established [34]. Likewise, the use of plant protein to stabilize an emulsion has been extensively studied in recent years, with soy, pea, and other lentil-based proteins being adopted by many studies to replace animal-sourced protein in emulsification [31,35]. Gumus et al. investigated the emulsification capability and stability of pea, lentil, and fava beans-stabilized emulsion under different pH, temperature, and salt conditions. In their study, lentil protein displayed superior stability across all environmental stressors [36]. Although the reason for lentils’ superior performance was not specified, it was suggested that extreme pH and salt content may have a more profound effect on the electrostatic repulsion of pea and fava proteins. Hydrophobic and steric interactions of lentil protein may also be responsible for the better temperature stability observed [36]. Recently, attention was also given to the use of globular protein fibrils over native plant protein isolates to improve emulsion stability and rheology. Micron-length protein fibrils are produced from native protein isolate via acid heat treatment at a pH lower than the isoelectric point of a protein [37]. Pang et al. investigated the use of rice bran protein fibrils to stabilize fish oil. It was shown that heating rice bran protein for 420 min achieved the highest emulsification capability with fish oil. This is attributed to an increase in the hydrophobicity of the fibrils and molecule flexibility at the interface [37].

### 2.2. Polysaccharide

#### 2.2.1. Structural Roles of Plant Polysaccharide in Emulsion Gel

Polysaccharides had been typically used as the structural component of an emulsion gel, both alone and in conjunction with protein in a mixed gel regimen. In a mixed gel, an emulsion is stabilized by a surfactant before the addition of polysaccharide and subsequent gelation. Similarly, the dispersed phase may act as both an active and inactive filler depending on the specific interactions between the selected protein/surfactant and the continuous polysaccharide phase [1,15]. A wide range of plant-based polysaccharides, such as methylcellulose, inulin, pectin, and native and modified starches, have been reported for use in emulsion gels to provide stability and alter the mechanical properties of the gels [38,39,40,41,42].

The gelation of polysaccharides may be induced by heat or by a coagulant (e.g., salt and acid) to form polymeric structures within the aqueous phase of an emulsion [15]. Particularly, interactions between the polysaccharide and the protein may provide enhanced performance over gels that use either component in a mixed gel. For example, the synergistic relationship between pea protein isolate (PPI) and inulin in an emulsion gel was studied by Xu et al. [39]. In their studies, when PPI and inulin were used together, a lower inulin content was required for gelation in contrast to pure inulin gel [39]. This was because inulin introduced more hydrophobic and hydrogen bond sites without adversely affecting protein electrostatic interactions, which greatly contributed to the firmness and improved properties of the gel [39]. Thus, the presence of synergetic interactions between proteins and polysaccharides was typically referenced for adopting a mixed gel regime [43,44,45].

**Figure 2:**
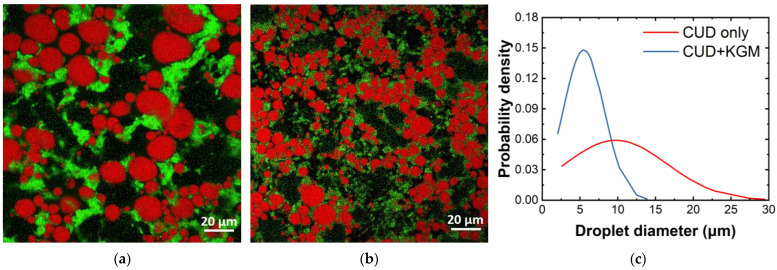
CLSM images of PPI and curdlan (CUD) emulsion gels with and without Konjac glucomannan (KGM). Both emulsion gels have identical total protein (5% *w*/*w*), oil (20% *w*/*w*), and total polysaccharide content (6% *w*/*w*): (**a**) Emulsion gel with 6% CUD; (**b**) Emulsion gel with 4.8% curdlan and 1.2% KGM; (**c**) Normal distribution of droplet diameters from (**a**,**b**). 50 droplets were randomly sampled from the respective images and processed using ImageJ^®^. Red: lipid fraction; green: protein and polysaccharide.

The rheological properties and stability of emulsion gels may also be modified through the addition of polysaccharides. Polysaccharides such as xanthan gum, guar gum, and potato starch were shown to increase the viscosity of emulsion gels in 3D printing, a structurally critical application [41,46]. In contrast to salt-induced soy protein emulsion gel, where the gel is too weak to hold a 3D structure, emulsion gels with xanthan and guar gums were able to hold structure with minimal loss in feature [46]. Additionally, increasing the viscosity of a gel during mixing may also result in smaller and more uniform droplets. Figure 2 shows confocal laser scanning microscopy (CLSM) images of PPI and curdlan gum heat-induced emulsion gel fabricated in our laboratory with or without konjac glucomannan (KGM), a plant-based polysaccharide. The emulsion gels in both images are identical in terms of total oil (20% *w*/*w*), PPI (5% *w*/*w*), and polysaccharide concentrations (6% *w*/*w*). Due to the high water absorption ability of KGM [47], viscosity was greatly increased in pre-gelled emulsion gel after its addition (data not shown). The two images only differ in polysaccharide composition, with Figure 2a showing gel with 6% curdlan and Figure 2b showing gel with 4.8% curdlan and 1.2% KGM. Droplets were larger and less uniform in curdlan-only emulsion gels compared to those with substituted KGM, as shown in the droplet size distribution in Figure 2c. The addition of polysaccharides that induce an increase in viscosity was seen to reduce the coalescence of the dispersed oil droplets during processing. Smaller droplet size is often associated with higher stability and gel strength in emulsion gels [48,49]. In addition, smaller droplet sizes may reduce oil release during gastric digestion and hence speed up gastric emptying [10].

Thermal stability of the gel structure may also be achieved using plant-based polysaccharides. This may be critical for certain applications as phase change may not be desirable at elevated temperatures. As such, KGM was used to create thermally irreversible emulsion gels for cooking stability [38,50]. Huang et al. [50] created a soy protein isolate (SPI) and KGM crosslinked composite gel that was able to maintain structure and texture after 20 min of cooking at 95 °C. Similarly, a combination of deacetylated KGM (DKG) and methylcellulose (MC) was reported by Jeong et al. [38]. Across all ratios between DKG and MC tested, all gels showed thermal irreversibility at 80 °C [38].

#### 2.2.2. Plant Polysaccharide as Emulsifier

Recently, interests have also pivoted to develop polysaccharide-only emulsion gels where polysaccharide is responsible for both emulsion stabilization and structure formation. Jiang et al. [51] explored the use of regenerated cellulose (RC) to stabilize a sunflower oil in water emulsion with curdlan, a bacterial gum, as the major structural component. The RC stabilized emulsion was shown to have high stability as no noticeable flocculation or separation was observed after 4 h of storage at room temperature and 1 h at 80 °C [51]. A plant-based emulsion gel was demonstrated by Zhou et al. [52] using polysaccharides extracted from psyllium husk. High stability was observed from the psyllium polysaccharide stabilized emulsion, as no visible phase separation was observed after prolonged storage at room temperature [52]. The reduction in polysaccharide content led to a softer gel as the polysaccharides were adsorbed at the oil/water interface rather than forming a continuous gel structure [52]. The psyllium gel was also found to have self-healing properties after the destruction of the gel structure due to the rapid re-formation of hydrogen bonds [52]. Moreover, psyllium husk is a prebiotic that promotes the health of gut microbiota and has been used to treat gastrointestinal diseases (e.g., constipation and diarrhea) [53]. The advantageous physical and bioactive properties displayed by polysaccharide-based emulsion gel in these two studies underline possible effective application of these gels in food and pharmaceuticals.

### 2.3. Lipid

The lipid phase in an emulsion gel is bound by a surfactant, and its interaction with the continuous phase is often guided through the surfactant. These dispersed droplets may be active or inactive, depending on whether the dispersed droplets directly interact with the continuous structural component. For instance, a non-ionic surfactant stabilized lipid phase is often deemed an inactive filler with little contribution to the gel structure. As a result, the presence of these droplets in a gel matrix impedes gel strength as it is analogous to porous structures [1,5]. Conversely, in protein-stabilized emulsion gels, a layer of protein is adsorbed to the surface of oil droplets. Interactions between the protein and the gel matrix in the continuous phase effectively make the lipid phase an integral part of the structure [1,5]. In practice, a variety of lipids have been exploited for their respective physical, chemical, and nutritional properties to deliver desirable product characteristics.

#### 2.3.1. Health Consideration on Lipid Selection

Most plant-extracted lipids exist as oils, with a higher proportion of mono- or poly-unsaturated fatty acids (MUFA and PUFA) than animal fat [54]. Since consumption of high PUFA or MUFA oils is linked to better cardiovascular health than saturated fat, oils extracted from canola, soybean, sunflower, and olive are the usual candidates for emulsion gels [55,56]. Naturally occurring plant fats such as coconut oil, cocoa butter, and palm oil were also commonly used in food due to their solid structure at room temperature and higher saturated fat content [54]. Moreover, the use of fully hydrogenated oil had emerged as an alternative to using natural plant fats, owing to its lower cost compared to natural plant fats while still being solid at room temperature [57]. This is in contrast to partially hydrogenated oils that contain trans-fat and have been proven to have worse cardiovascular outcomes [57].

#### 2.3.2. Enhancement of Textural Properties by Lipid

Aside from health benefits, the choice of lipid was also typically linked to the texturization of the final gel [15]. Oil types were found to have profound effects on the rheology and mechanical properties of a gel. Gels containing solid plant fat are typically stiffer and harder than those that use plant oil. Gu et al. [58] analyzed the effect of different oil types on the SPI emulsion gels. In their study, sunflower oil, soy oil, and palm stearin were emulsified and gelled in an SPI matrix. Palm stearin gel was a stiffer and harder gel than both of its oil counterparts, independent of the gelation method (GDL and heat) [58]. It was believed that palm stearin’s crystalline structure allowed for the creation of an effectively absorbed protein layer around the dispersed droplets during emulsification, which increased the rigidity of the gel [58]. In more specific applications such as animal fat analog, Jeong et al. [38] showed that the use of coconut oil often resulted in superior hardness, springiness, and chewiness in textural analysis over canola oil at identical gel formulation at room temperature. Samples prepared using coconut oil showed a difference of up to 3.6 times in hardness over canola oil at 25 °C, with differences diminishing as the two samples were heated up to 80 °C [38].

#### 2.3.3. Lipid as a Carrier of Nutrients

The lipid phase has also been used as a carrier of lipophilic additives to create functional foods using emulsion gel to increase the bioaccessibility and stability under heat and light of bioactive compounds [10]. Common plant oils, including corn, flaxseed, and soybean oil, are seen as carriers of bioactive ingredients ranging from vitamins to phenolic compounds such as cholecalciferol, β-carotene, and curcumin in plant-based emulsion gel [59,60,61]. In these studies, no indication was observed that a specific oil type was selected based on its functional attributes or suitability as a carrier.

Regardless, recent studies indicated that solid fat content (SFC) and oils high in antioxidants may increase the stability of the loaded material in addition to solubilization. In a whey protein isolate (WPI) and coconut oil emulsion gel created by Lu et al. [62], β-carotene was found to be more stable in an emulsion gel that has a higher SFC in both UV treatment and elevated temperature storage (55 °C). This was attributed to the inability of free radicals to travel through solid fat and the higher stability of coconut oil compared to other oils (e.g., corn), as well as the antioxidative ability of the medium-chain triglyceride (MCT) component in coconut oil [62]. The additional antioxidative effects of MCT in coconut oil were also demonstrated in an emulsion designed for lycopene delivery, where lycopene retention was approximately 40% better at 37 °C storage after 2 weeks than that of other oil types (e.g., sesame, linseed, and walnut oil) [63].

## 3. Processing of Plant-Based Food Emulsion Gels

As the applications of emulsion gels expand to encompass a growing variety of functions, diverse processing techniques and configurations have been proposed to achieve the desired food-like properties. These promising approaches are employed for the processing, transportation, and targeted release of food additives, functional ingredients, and bioactive substances, offering flexibility in tailoring food disintegration and sensory properties, as well as structural and functional parameters. The processing of emulsion gels is important due to their potential as fat substitutes in various animal-based products. For example, these emulsions can enhance the nutritional properties of meat products, as they are more adept at transporting and safeguarding oxidized lipids in food while also preserving flavor compounds and bioactive compounds. Soft or hard-textured emulsion gels are preferred for fat replacement over conventional emulsions without gel formation. It can better imitate the physical attributes of animal fats (lard), such as texture and water-holding capacity. Emulsion gel is formed using various technologies such as high internal phase, ultrasonication, high-pressure homogenization, and microfludization [64].

In a study on the formation of protein-based emulsion gel (yogurt and tofu analogs) using fava beans, whole nutrients were utilized [64]. Fava bean was processed through thermal pre-treatment, dehulling, milling, plant oil addition, homogenization, starch gelation prevention, and finally, the inducement of protein gelation. Starch removal and hydrolysis were used to prevent starch from gelation [64]. Starch hydrolysis showed better results in yogurt analog production, as this process increased the gel’s strength and viscosity. In addition, hydrolysis utilized the whole floor with no waste production. Moreover, the tofu analog was better prepared when formed with starch removal, as opposed to starch hydrolysis, because it decreased the gel strength and water-holding capacity of both products. These two methods are preferred to generate protein-based emulsion gel from whole fava bean flour.

**Figure 3:**
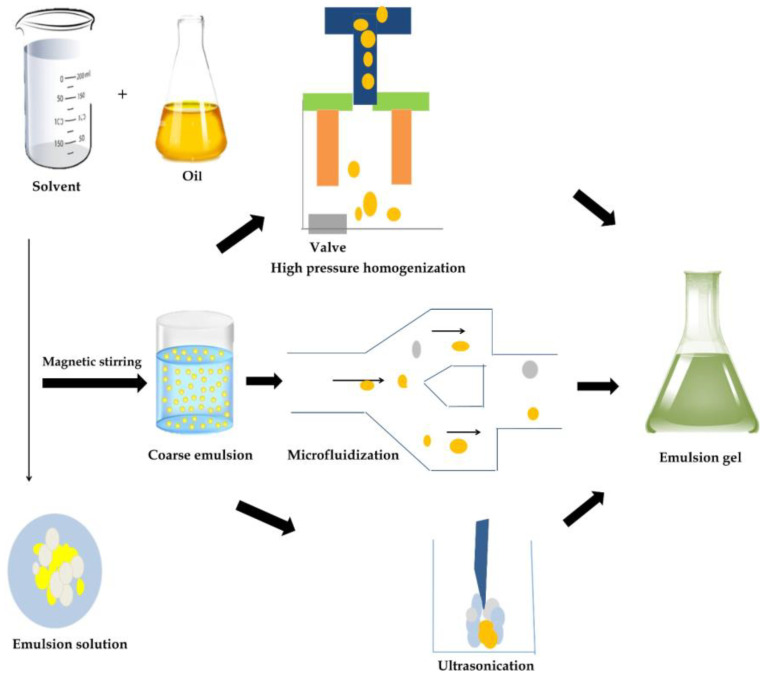
Formation of plant-based emulsion gel.

In many studies, the fabrication of an emulsion gel generally begins with high-shear mixing between the oil phase and aqueous phase, where the surfactant and or biopolymer are solubilized or suspended [65]. Although high-shear mixing is generally straightforward, other methods have been proposed to further improve stability and modify the final gel properties [66,67]. This includes high-pressure homogenization (HPH), ultrasonication (UF), and microfluidization (MF). Various emulsion gels can be prepared using these methods and exhibit differing gel structures and interactions among droplets with an impact on mechanical and release characteristics. Figure 3 illustrates the preparation of plant-based emulsion gel through high-pressure homogenization, microfluidization, and ultrasonication.

There are three main processing methods for producing emulsion gels in the food industry, which are HPH, US, and MF. HPH involves flow restriction, which produces greater pressures and shear forces (up to 350 MPa). However, sometimes the restriction of flow generates larger and non-uniform particles due to pressure fluctuation from the higher pressure. This is in contrast to MF, which can only exert a maximum of 275 MPa. The fixed geometry of the MF instrument ensures constant pressure delivery and equal size distribution [68], while ultrasonication works based on the principle of cavitation, which is produced through mechanical vibration. These processing methods are applied to the covert dispersed phase of the emulsion into fine, tiny droplets for better gel characteristics such as water-holding capacity, storage modulus, gel strength, and stability [69].

### 3.1. High-Pressure Homogenization

High-pressure homogenization (HPH) is an emerging technique with a pressure pump that exerts pressure up to 200 MPa [70]. It is applied for fluid stability, protein and polysaccharide modification, and improving the rheological characteristics of emulsion gels [71,72]. Alvarez-Sabatel et al. [19] evaluated the gelling characteristics of inulin after HPH application at 103, 207, or 296 MPa at an inlet temperature of 3.5 ± 1 °C for 5 min. The HPH treatment lowered the minimum concentration requirement necessary for gel texture and enhanced the crystallization behavior of inulin due to droplet dispersion, while the high pressure of 296 MPa reduced water retention efficiency and hurt the gel structure. Additionally, the inlet temperature was enhanced and reached a maximum (33.51 ± 0.53, 49.99 ± 0.96, and 61.67 ± 0.85 °C) due to the increase in pressure. Moreover, hazelnut beverage samples were subjected to HPH at a pressure of 150 MPa and a concentration of 10 g 100 mL^−1^ at 26–38 °C with a GDL acidification (2 g 100 mL^−1^ at 150 min), and the strength, rheological, and textural properties of cold-set gels were investigated. It increased the viscosity of hazelnut gels and improved the protein structure and gel characteristics with acidification [73].

The impact of thermal treatment on the rheological properties of SPI gels was investigated with acid-induced gelation and further treated with HPH at 400 bar pressure. The results showed that thermal treatment (95 °C, 20 min) improved the mechanical properties, and HPH increased the viscoelastic properties of the gel [74]. Furthermore, the HPH effect on the functional characteristics of SPI emulsion gel was evaluated at different pressures (from 5 MPa to 80 MPa), showing an increase in get strength (G’ = 291 Pa to G’ = 528 Pa at 80 MPa) and water holding capacity (WHC) (87.7% to 91.4%) at 5 to 20 MPa and constant values from 20 to 80 MPa pressure. Overall, the HPH-treated gel has a more uniform network [75]. The potential of using HPH to produce potato protein isolate yogurt alternatives with low and high oil contents has been evaluated at various oil concentrations (1.5, 3, and 10%) and homogenization pressures (0.1 MPa, 30 MPa, and 200 MPa). The HPH decreased the emulsion particle size in comparison to untreated samples. Moreover, the emulsion whiteness index had increased. The greatest value of the whiteness index (76.01 ± 0.50) was obtained at 200 MPa with 10% oil content, while the least (65.33 ± 2.05) was observed at 0.1 MPa with 3% oil content. The creaming velocity at 3% oil concentration was reduced from 10.70 (0.1 MPa) to 0.59 (200 MPa). In the end, gels showed smaller oil droplets and finer constituent distribution [76].

### 3.2. Ultrasound

Emulsion gel structure and functional properties have been significantly affected by homogenization. Ultrasound homogenization (UH), a safe, economical, and environmentally friendly technique, comprises acoustic waves with 10–1000 W/cm^2^ power and 20–100 kHz frequency and produces mechanical and shearing effects through cavitation [77]. Various research has proven that the UH enhanced the protein properties such as aggregation size and solubility, thus increasing protein adsorption at the interface [77,78,79]. Moreover, the UH caused unfolding and an improvement in protein bonds at the interface, improving the gel’s properties [77].

For instance, in a study by Aliabbasi et al. [78], the impact of high-intensity ultrasound on pinto bean protein isolate (0, 25, and 50 min at 200 W) was evaluated, and the gelation process was conducted using GDL. The key findings showed that the intrinsic fluorescence and the structure of the gel were improved. Moreover, the WHC and gel strength were increased, and the gel was inoculated with curcumin. Additionally, cross-linking ability decreased the swelling ratio of the gel and changed the curcumin release rate from the emulsion gel [80]. Furthermore, SPI and pectin emulsions were produced by heat treatment, and the effects of ultrasound on the texture, gel characteristics, and emulsion stability were assessed at various powers (0, 150, 300, 450, and 600 W). X-ray diffraction and Fourier transform infrared spectroscopy (FTIR) demonstrated that the interactions between SPI and pectin were improved due to the increase in hydrogen bonding and altered the crystallization of emulsion gels. Moreover, the droplet size was decreased, and WHC was increased by increasing the power (450 W) and denaturation temperature (128.2 °C to 131.9 °C). However, the bioaccessibility (82%) and chemical stability (78.3 ± 2.0%) of β-carotene were improved [81] due to the decrease in the aggregation of oil droplets and degradation of β-carotene during the digestion of the emulsion gel.

In a study by Mozafarpour and Koocheki [82], emulsion gels were produced using grass pea protein isolates (GPPI) at various ultrasonic treatment conditions (amplitudes of 25, 50, and 75% for 5, 10, and 20 min). Emulsion gel formation was stimulated by transglutaminase and stabilized by sonication. The first step, which was called the “fast step,” caused weak gel formation, then the second, referred to as the “slow step,” increased the gel strength. The results showed uniformity in particle distribution and improved velocities of emulsion gels. A harder emulsion gel was produced at 75% amplitude for 10 min. The WHC and mechanical properties were improved. Ultra-sonicated GPPIs had a fine microstructure compared to untreated GPPIs. Ultrasonication was applied in the formation of emulsion gels for topical drug delivery of metronidazole at 20–25 kHz at 150 W. Sorbitan monostearate (SMS) was used as a stabilizer at the sesame oil/water interface. Emulsion gels were prepared with various proportions of SMS ranging from 2.5–10% (*w*/*w*), while the water proportion ranged from 20% to 80% (*w*/*w*). The emulsion gel’s viscosity and firmness had been increased by sonication processing. The drug-loaded gels exhibited antimicrobial efficiency, showing potential as a carrier for drugs [83]. Geng et al. [77] prepared soy protein bulk emulsion gels incorporating CaCl_2_, GDL, and transglutaminase with the UH treatment (40% amplitude, 20 kHz, 3 min) for β-carotene delivery, showing improved bioaccessibility of β-carotene in bulk emulsion gels. It was noted that β-carotene bioaccessibility was increased when encapsulated in ultrasound-treated emulsion gels (82.39 ± 0.02%) compared to other emulsions (63.37 ± 0.09%). Moreover, findings suggested that WHC (99.81 ± 0.19%) and gel strength were improved (91.02 ± 3.58%) in transglutaminase-induced samples compared to CaCl_2_ and GDL-induced samples. Overall, it can be suggested that ultrasonication improved WHC and the strength of the gel sample.

### 3.3. Microfluidization

Microfluidization (MF) is a high-energy processing technique in which pressure is applied to force the liquids through micro-channels, thus helping in emulsification by the synergistic effects of shear forces and cavitation [84]. It helps overcome issues such as large particle size and emulsion instability [85]. The MF was applied to the pea protein emulsion (5% pea protein and 50% sunflower oil) at 50 MPa for one pass, which caused the cold-set gel formation. The findings showed that MF increased gel strength, decreased particle size, and improved viscosity with an increase in pressure [86]. In a study by Yang et al. [87], γ-zein from corn was produced into particles to prepare gel-like emulsions. The MF was performed at 0.1 to 120 MPa to assess the rheological properties and structure formation of the emulsions. MF reduced particle size as well as gel network by droplet clusters as shown in microscopy where γ-zein particles provided stabilization, and the excess protein provided particle network. With the increase in pressure, gel strength increased due to the formation of more hydrophobic interactions and disulfide bonds. The results showed that emulsions prepared at 0.1 MPa have a weak gel structure, while emulsions prepared at 120 MPa have a stronger gel structure and higher stability.

Furthermore, MF was applied at 50, 70, and 130 MPa to native pea (NP) and soluble thermally aggregated (SA) pea globulin-based emulsions at neutral pH. Emulsions were assessed for their properties such as protein adsorption ability, charge emulsifying, flocculation, and creaming stability. It showed that NP and SA-based emulsions were more flocculated and had a coarse appearance. The MF pressure reduced the flocculation size when the pressure was increased due to the processing of emulsification. The NP-based emulsion creaming stability was decreased, while the SA-based emulsion creaming stability was improved as the pressure increased due to a decrease in flocculation size and thus the formation of a gel-like structure. The key findings suggested that MF could be used to improve emulsification properties as it reduced particle size, increased physical stability, and improved the viscosity of the emulsion gel [88].

## 4. Characterization of Plant-Based Food Emulsion Gels

To characterize plant-based food emulsion gels, recent research has mainly focused on these properties: appearance, rheology, texture, microstructure, and stability. Investigations into these properties provide insights into the behavior and overall quality of the emulsion gel, which could optimize the formulation and processing of plant-based emulsion gels. An overview of the characterization techniques of plant-based emulsion gels is presented in Table 1.

### 4.1. Appearance

Visual inspection as a direct method is commonly used in research to rapidly obtain preliminary information on the samples, of which color, gel fabrication (viscoelasticity, compactness, hardness), and stability/instability (phase separation, creaming, aggregation, coalescence) were the most relevant. These results were often discussed with other characteristics to support the conclusion from the perspective of micromorphology [89,90,91,92,93,94,95].

Color is recognized as an attribute that mostly affected appearance as perceived by consumers directly. Besides visual inspection, color can also be quantitively characterized by CIELAB color space coordinates (lightness, L*; green/redness, a*; and blue/yellowness, b*) by using a colorimeter [96] or image analysis software (for example, ImageJ^®^ software), which can convert digital images to coordinate data [97].

### 4.2. Rheological Properties

**Figure 4:**
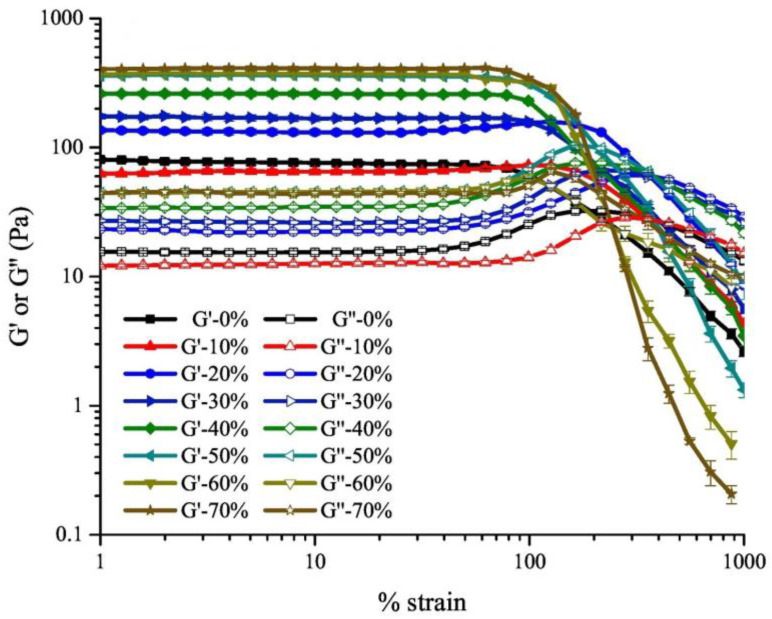
Large-amplitude oscillatory shear behavior as a function of strain amplitude for a novel starch-based emulsion gel with different oil volume fractions (0–70%). Adapted from Ref. [95] with permission from Elsevier.

Rheological properties were measured to characterize the flow and deformation properties of plant-based emulsion gels under applied stress. Rheology measurement is important for understanding the behavior of the gel during processing, storage, and consumption, which is the foundation of optimizing the formulation and processing of the materials. The storage modulus (G′) and the loss modulus (G″) were used to indicate the elasticity (solid-like) and viscosity (liquid-like), respectively. The loss factor (tan δ) was commonly used to indicate the gel-like status of the sample [82,89,95,103]. Usually, a strain sweep test would be used to identify the linear viscoelastic region (LVR). Figure 4 shows an example of research on a starch-based emulsion with different oil volume fractions ranging from 0 to 70%. The transition from a linear to a non-linear viscoelastic region in a strain sweep graph is shown, in which the horizontal-like region was defined as the LVR. The yield stress (σ_c_) refers to the stress causing the first non-linear deformation, which was used to reflect the strength of the emulsion gel in many research [91,92,93].

Small amplitude oscillatory shear (SAOS) within LVR was commonly used in the characterization of the viscoelastic properties of plant-based emulsion gel. A frequency sweep test was used to indicate the fabrication of a gel-like structure (G′ > G″, both frequency independent). The creep-recovery test was used to study transient viscoelastic behavior [95,98,99]. A time sweep test (gelation kinetics) was used to investigate the change in gel texture at a certain frequency and temperature over a period of time [40,42,98]. Moreover, a time sweep has been used to monitor the enzyme performance in terms of gelation induced by crosslinking with transglutaminase in plant-based emulsion gel [82,100].

A large amplitude oscillatory shear (LAOS) test has been used to investigate the rheological behavior while the equilibrium is lost and the intermolecular bonds are (partly) broken down. LAOS can provide more information about the structure evolution under large deformations (e.g., 1–1000%), which is meaningful in industrial applications. Several research works on plant-based emulsion gel used the LAOS test to gain insight into the texture and structure properties, in which crossover strain, phase angle, Lissajous curves analysis, Fourier-transform, and Chebyshev coefficients analysis were further revealed [92,95,101]. The utilization of the LAOS test is less reported in the current literature. However, this technique has caught considerable interest in recent years as a valuable tool for the characterization of the viscoelastic behavior of plant-based emulsion gel [92,95,101].

### 4.3. Texture

**Figure 5:**
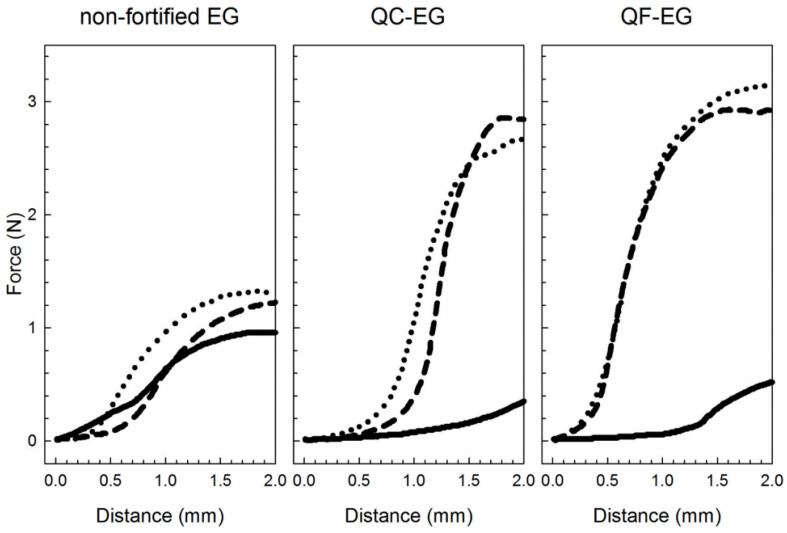
Force vs. distance plots obtained from penetration tests of non-fortified (**left**), QC-fortified (**middle**), and QF-fortified (**right**) soft emulsion gels (EGs) stored at 4 °C for 1, 14, and 28 days (solid, middle dashed, and dotted line styles, respectively) (QF: quinoa flour; QC: quinoa concentrate). Sodium alginate, sodium citrate, CaCl_2_, and GDL in EG systems used were 10 g/kg, 100 mmol/L, 75 mmol/L, and 30 g/kg, respectively. EGs were prepared by high-speed homogenization (25,000 rpm for 1 min) of the aqueous dispersion and olive oil phases in a mass ratio of 3 g:1 g, respectively. Adapted from Ref. [97] with permission from Elsevier.

The texture measurement is important for the sensory experience and overall quality of a plant-based emulsion gel. It can help optimize the formulation and processing of the material to achieve desirable textural characteristics. As reported, texture profile analysis (TPA) is usually performed by a texture analyzer. The penetration test or double-compression tests were usually used to characterize the gel strength of the material. For a penetration test, a sample was penetrated axially at a certain constant speed by a probe, and a force vs. distance plot was obtained. The firmness (FF) was then determined as the initial slope of the penetration profiles [40,82,97,99]. An example is provided in Figure 5, which utilizes the penetration test to study and compare the emulsion gels with different compositions stored under specific conditions for different periods. The results demonstrate significant differences between the various emulsion gel samples, indicating variations in their textures.

For a double-compression test, the samples were subjected to two consecutive deformation cycles over a predetermined distance at a certain constant speed by a probe. A force vs. time plot was obtained, from which parameters such as hardness (N), springiness (mm), gumminess (N), chewiness (N), cohesiveness, viscidity (J), and stiffness (N) [42,93,97,103] can be determined. Additionally, these original parameter data can be normalized to one-dimensional data based on Minkowski distance. Zhang et al. [102] have normalized the three-dimensional data (hardness, springiness, and cohesiveness) into one-dimensional data, which is defined as a comprehensive property index (CPI). This CPI reflected the overall texture properties of the emulsion gel material and was used for further discussion in the relationship between textures and gelation induction methods of emulsion gel.

### 4.4. Microstructure

#### 4.4.1. Droplet Size Distribution

Droplet size distribution was determined to understand the size distribution of the dispersed phase in plant-based emulsion gels. The droplet size distribution has an impact on the stability, rheology, and sensory properties of the materials [100]. Several techniques were used to measure the droplet size of plant-based emulsion gel, such as laser diffraction [91,94,100,104,105], dynamic light scattering (DLS) [82,92,98], and microscopy (discussed in Section 4.4.2). Additionally, DLS can also be used for the size measurement of protein aggregates. Wang et al. [98] conducted research in which they used DLS to measure the size of soy protein aggregates in a soy oil emulsion.

#### 4.4.2. Microscopy

Microscopy techniques were used to visually inspect the morphological properties of plant-based emulsion gel on a micro level. Some frequently used techniques are optical microscopy (OM), polarized light microscopy (PLM), confocal laser scanning microscopy (CLSM), scanning electron microscopy (SEM), and scanning electron cryo-microscopy (Cryo-SEM).

**Figure 6:**
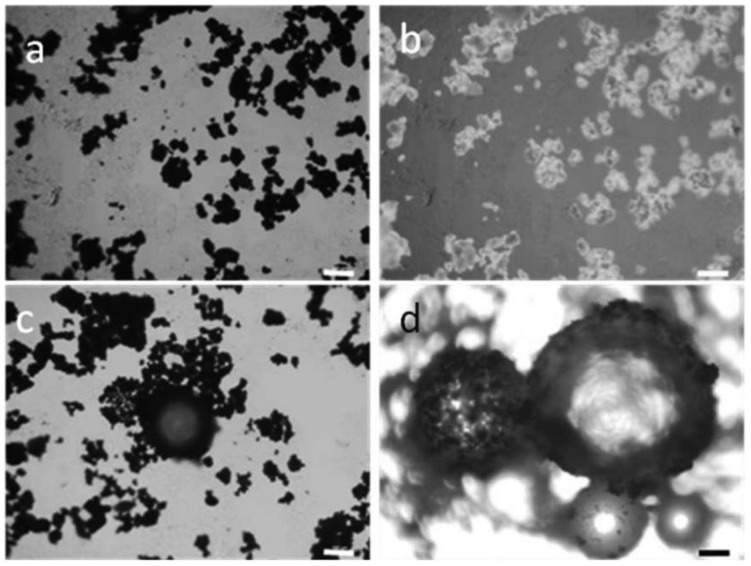
Typical bright-field optical (**a**,**c**,**d**) and polarized optical micrographs (**b**) of air-dried amorphous cellulose (**a**,**b**), and styrene Pickering emulsion (**c**,**d**) stabilized by amorphous cellulose and polymerized using AIBN (azobisisobutyronitrile) as initiator. The scare bar is 100 mm. Adapted from Ref. [89] with permission from Elsevier.

Optical microscopy (OM), also known as light microscopy, is a technique that has been used to investigate the microstructure of emulsion gel samples. With this technique, it is possible to visualize the size and organization of fat droplets [89,104,107]. A schematic principle of a classical optical microscope is shown in Figure 5 [106]. The polarized light microscope (PLM) was used in some research on plant-based emulsion gel [89,92,105]. This technique can provide additional information about the sample’s structure and composition through the use of polarized light, which is affected in specific ways when passing through specific materials, such as crystals, fibers, and starch. Examples of the OM image and the PLM image are shown in Figure 6, from which a styrene-in-water emulsion stabilized by amorphous cellulose was observed. The OM image (a, c, and d) can provide information about the styrene droplet size, shape, and organization. The PLM image (b) can provide information about the cellulose behavior in the system.

Confocal laser scanning microscopy (CLSM) uses a focused laser beam to illuminate a sample, and only the in-focus light emitted from the sample is collected, resulting in 3D images with a high resolution [108]. According to reported research, the lipid phase was usually stained by Nile blue, and proteins were usually stained by Nile red or rhodamine B. The use of CLSM in studies on plant-based emulsion gel can reveal information about lipid droplets and protein behavior. For example, it can determine the lipid droplet size and distribution, the existence of aggregation, and the affinity of proteins for the interface. This information can provide insights into the structure, strength, and unification of the emulsion gel [75,93,97,98,99]. An example of a CLSM image can be found in Figure 2.

**Figure 7:**
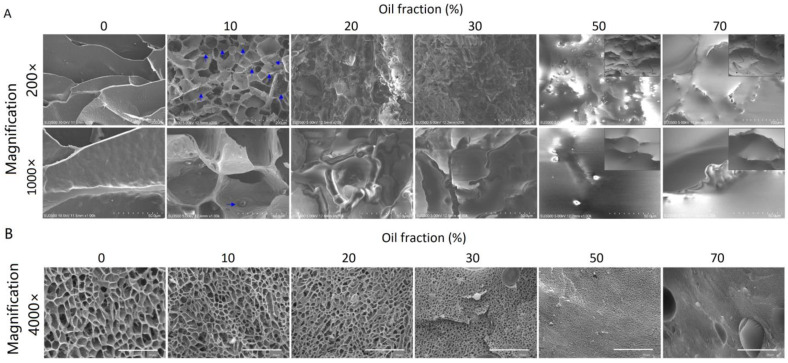
(**A**) SEM micrographs of the emulsion gels with 0–70% oil fractions and the corresponding Cryo-SEM micrographs of the emulsion gels with 50% and 70% oil fractions (located in the upper right corner of the SEM micrographs): The blue arrows in the 10% oil sample indicate the oil droplets; the Cryo-SEM micrographs of 50% and 70% oil emulsion gels were taken because these samples were blurred under SEM observation due to the high content of oil. (**B**) Cryo-SEM micrographs of the emulsion gels with 0–70% oil fractions. Adapted from Ref. [95] with permission from Elsevier.

Scanning electron microscopy (SEM) uses a focused electron beam to scan the sample surface, producing high-resolution images by detecting secondary electrons emitted from the sample. This technique can provide more detailed information about the network structure of emulsion gels, such as pore size and distribution, wall thickness, and oil droplet distribution [40,95,102]. Freeze drying is a common method for sample dehydration. However, dehydration by ethanol was also reported in the research by Wang et al. [98]. Scanning electron cryo-microscopy (Cryo-SEM) is another similar microscopy technique in which a pre-freezing by liquid nitrogen is performed to maintain the structure of emulsion gels. The frozen samples are maintained at very low temperatures (e.g., −70 °C) while imaging [95]. An example of SEM images and Cryo-SEM images is shown in Figure 7. For the same sample, the image from SEM was blurred due to some alterations in their morphologies, resulting from the high oil content. While from Cryo-SEM, the original structures were well maintained.

### 4.5. Stability

The stability study of plant-based emulsion gels is important to maintain their characteristics over time. This study can provide information on the factors that affect gel stability, which can be used to optimize the formulation and processing of the materials. The stability of the plant-based emulsion gel can be reflected in multiple aspects, such as the properties mentioned previously on appearance, rheological properties, texture, and microstructure (Section 4.1, Section 4.2, Section 4.3 and Section 4.4). Thermal properties and the zeta potential were measured to quantitatively characterize the gel stability, while freeze-thaw cycling tests and centrifugation tests were used for stability study in the reported study.

#### 4.5.1. Thermal Properties

**Figure 8:**
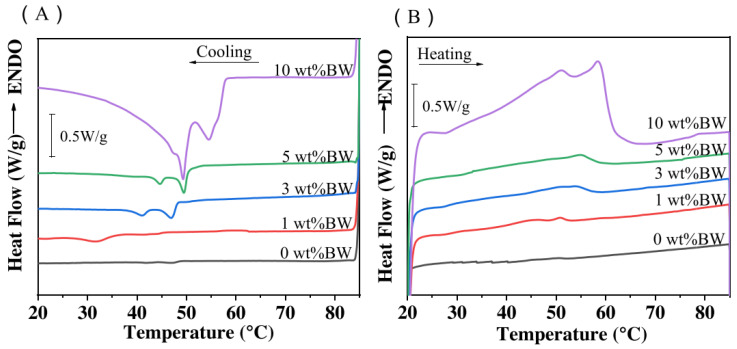
DSC thermographs of low-oil emulsion gels with 0, 1, 3, 5, and 10% (*w*/*w*) Beeswax during the cooling (**A**) cycle at −5 °C/min and the heating (**B**) cycle at 5 °C/min. Adapted from Ref. [92] with permission from Elsevier.

The thermal stability of plant-based emulsion gel has been reported as having important implications for sensory and physical properties, as well as processing and storage characteristics. Differential scanning calorimetry (DSC) is a commonly used technique to characterize the thermal properties of plant-based emulsion gel, from which the amount of heat required to increase the temperature of a sample is measured as a function of temperature. Thermal information such as the on-set and maximum temperatures of phase transition, the temperature for protein denaturation, and their respective enthalpies can be obtained from the DSC curves. Research conducted by Liu et al. [92] has used this technique to monitor rapeseed oil solidification in emulsion gel formation (Figure 8). The behavior of the intermolecular interaction, small aggregate disruption, and protein denaturation were also investigated in the reported research on plant-based emulsion gel [75,82]. Thermogravimetric analysis (TGA) is another common method to characterize the thermal properties of a plant-based emulsion gel, in which the mass of a sample is measured over time as the temperature changes. Thermal information, such as the thermal degradation temperature of emulsion gels, can be obtained to indicate the gel’s thermal stability [42].

#### 4.5.2. Zeta Potential

**Figure 9:**
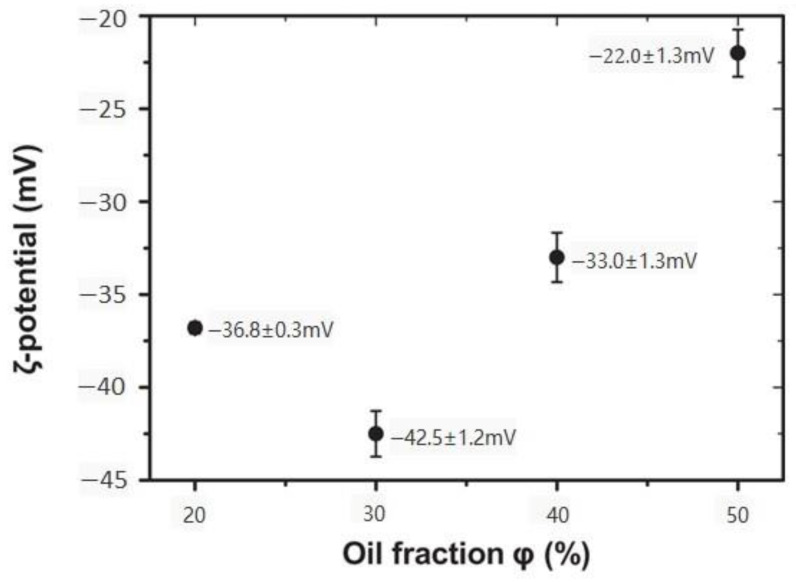
Zeta potential of PPI-stabilized emulsions with different oil-weight fractions (φ). Adapted from Ref. [100] with permission from John Wiley and Sons.

The use of zeta potential measurement by an electrophoresis instrument in the characterization of plant-based emulsion gel stability has been reported. The zeta potential is the electrostatic potential at the slipping plane, where the continuous phase (typically aqueous) begins to flow at a small distance from the droplet surface. Zeta potential measurements can indicate the interaction between droplets. Generally, a larger absolute value of zeta potential reflects a more stable emulsion system. This value can be influenced by the oil-weight fractions (Figure 9), droplet size, the amount of attached protein on the interface, and the denaturation and unfolding of proteins as reported in [82,100].

#### 4.5.3. Water Holding Capacity (WHC)

The pores in the gel network can provide space for additional water. WHC measurement can provide insights into the microstructure and functional properties of emulsion gels [42,75,82,98,100]. It is generally accepted that a higher value of WHC implies the formation of a gel network with higher strength and a more uniform structure, resulting in a stronger ability to retain water molecules.

#### 4.5.4. Freeze-Thaw Stability

Freeze-thaw cycling was reported as a method to assess the stability of plant-based emulsion gel under temperature stress [14,90,103]. Briefly, this method involves subjecting the emulsion gel to repeated freezing and thawing cycles, with the fluid loss being measured after each cycle. Freezing destabilizes an emulsion by promoting flocculation and coalescence as water and lipid crystalize [109]. The structural change is then made apparent as the gel is thawed, where phase separation will be observed. The formulation, including the type of oil used, greatly influences freeze-thaw loss and the emulsifier used [109].

## 5. Potential Applications of Plant-Based Emulsion Gels in the Food Industry

The prevalence of emulsion-based foods in the everyday diet has created immense opportunities for research and development in replacing current animal-based foods with plant-based mimics. Even though annual sales of plant-based food had grown by 54% from US$4.8 billion to US$7.4 billion in 2021, plant-based milk remains the largest sector of sales [110]. Although other plant-based dairy products make up the second largest sector, the contribution of other key areas, such as plant-based meat and egg alternatives, remained minor [110]. This may be due to the challenges involved in creating plant-based alternatives that can effectively replicate the flavor, texture, and mouthfeel of animal-based food products such as meat and dairy products. Therefore, emulsion gels, in whole or as an ingredient, were thought to be able to improve some of these limitations. A summary of selected studies on possible plant-based emulsion gel application is presented in Table 2.

### 5.1. Dairy Alternatives

Emulsion gels formed by dairy proteins such as whey and casein were typically described as model emulsion gels [5]. However, the key textural characteristics of casein gels, such as those seen in yogurt and cheese, have been difficult to replicate by plant protein alone [18]. This is due to differences in plant protein structure, namely the lack of random coils and phosphate groups that could be linked by the presence of calcium ions [18]. Thus, to create convincing analogs of dairy products, emulsion gels of various formulations have been proposed.

The development of plant-based yogurt has seen some success in emulating dairy yogurt in both research and commercial products. [26,111,112,119]. However, to design a successful plant-based yogurt-like product, further insights may be drawn from the literature to determine critical factors in formulation and processing. Several studies have indicated that the sensory attributes (taste, aroma, texture, and appearance) of plant-based emulsion gel for yogurt purposes depend on the starter culture used [112,120]. In a previous study for pea protein fermentation, Ben-Harb et al. [112] used a microbial consortium design and found a reduction in undesirable odors from the use of legume-proteins after fermentation, as “smoked” and “coffee” notes were better identified by panelists than “cut grass” notes. Aside from full-fat yogurt, HPH treatment of plant protein yogurt may be used to create plant-based low-fat and Greek-style yogurt [76]. Rheological and textural characteristics were shown to be tunable at different pressures and oil contents without changing the formulation [76]. Thus, as studies into interactions between yogurt components are further advanced, a successful yogurt-like product may be created using plant-based emulsion gel.

On the other hand, emulsion gel was also employed to create plant-based cheese analogs of various types, including soft, semi-hard, and spreadable cheeses [96,113,114,121,122]. In this regard, several important properties of plant-based cheese analogs were identified by Grossmann and McClements [121], such as texture, meltability, shreddability, and aroma. The recreation of texture in plant-based cheese analog broadly follows other similar applications that require texturization. Ferawati et al. [113] trailed the creation of a semi-hard cheese analog using various pulse proteins, canola oil, and κ-carrageenan. In their experiments, fava bean flour was most suitable to mimic the texture of Gouda cheese with comparable characteristics that may be further improved with optimization of processing conditions and protein ratios [113]. In terms of meltability, recent advances in zein protein-based cheese formulation with xanthan gum and starches (tapioca and corn) have shown promising results in creating cheese analogs with similar characteristics to Cheddar cheese [114]. Zein protein gel was shown to better trace cheddar cheese than PPI-based analog, gluten-based analog, and commercial plant-based cheese at 30% (*w/w*) protein content [114]. Nevertheless, as pointed out by others, a detailed sensory analysis would be required to provide a holistic analysis of the formulation’s suitability as an equivalent of a given cheese type [123]. This may be especially true as plant-based cheese analogs were only able to achieve comparable meltability at high protein and low oil content, which may impact the mouthfeel of a product.

### 5.2. Meat Alternatives

Emulsion gel is often designed as an “animal fat analog” (AFA) to mimic animal fat tissue. Emulsion gel-based AFA has seen use as the primary fat component in alternative meat products or as fat replacers in animal meat products to improve the nutrition profile of these products [56]. In the current commercial plant-based meat products, the fat component is often represented using unstructured plant fat, such as coconut oil or cocoa butter [124]. Although unstructured fat may be present as fat marbling in a raw and chilled state, naked plant fat often lacks texture, especially after cooking [115]. This is further compounded by possible processing difficulties during extrusion and loss of appeal due to smearing if mishandled [125,126]. Hence, emulsion gel was proposed as a potential solution.

Emulsion gels for AFA had been created using various gelation methods in both the protein-only gel and mixed gel forms. For this application, qualities such as appearance, texture, and thermal performance are parameters that were widely measured. Dreher et al. [115] created a soy-based emulsion gel crosslinked by transglutaminase with a plant fat and oil blend. Although the hardness observed was lower compared to pork fat tissue, the introduction of solid fat content enabled tunable characteristics in crosslinked emulsion that may imitate the melting characteristic of fat in animal tissue [115]. A transglutaminase crosslinked soy protein and KGM gel using coconut oil were proposed by Huang et al. [50]. Emulsion gel at 5% SPI (*w*/*w*), 4% KGM (*w*/*w*), and 10% (*w*/*w*) oil was found to have similar hardness and springiness values to pork back fat. The colorimetry of emulsion gel samples was also found to be able to mimic pork back fat in terms of lightness (L*) and blue/yellow (b*) [50]. Both characteristics demonstrated promising results in recreating animal fat using plant-based emulsion gel. Furthermore, the stability of emulsion gel during and after cooking was demonstrated by several studies [38,41,50]. Thermal irreversibility and cooking profiles may be modified by the selection of polysaccharides or the introduction of additives [38,41]. However, a good balance between each characteristic, including additional parameters such as health and palatability, is yet to be found at present.

### 5.3. Egg Yolk Alternatives and Baked Goods

Egg yolk protein has an extensive role in food as an emulsifier and thickener in condiments and confectionaries [127]. Notably, in mayonnaise analogs, statistically indifferent textural, extrusion, and sensory properties were seen in chickpea protein stabilized mayonnaise compared to egg-based mayonnaise at 70% oil content [117]. In other formulations, particularly in HIPE, it was shown that the textural and rheological characteristics of gels are tunable based on biopolymer content [118,128]. Such a characteristic allows for the formulation of low-fat mayonnaise, which maintains similar textural characteristics.

Similarly, an emulsion gel-based fat replacer was also used in baked goods to deliver a better nutritional profile. Inulin and extra virgin olive oil emulsion gel were used as a partial replacement for butter in shortbreads [116]. However, the lower plasticity of emulsion gels compared to butter was deemed problematic as it was not able to entrap gas released by the leavening agent. Along with a lower spread during heating and limited gluten formation, a harder product was obtained [116]. The observation revealed that additional processing may be required for emulsion gel, such as the incorporation of air within the gel matrix, for better uses as a fat replacement in baked goods, especially for short doughs.

### 5.4. Functional Foods

As previously indicated, emulsion gels may be used as a controlled-release regimen to protect and deliver nutrients or other active ingredients into the human body. Emulsion gels have been proven to protect active ingredients against ultraviolet light, free radicals, and temperature [61,62,93]. Both the lipid and aqueous phases could act as carriers of ingredients. Ghialdi et al. [59] developed an emulsion gel-based confectionary using inulin, pectin, and gum Arabic to protect and deliver vitamin B_12_ and D_3_ in their aqueous and lipid phases, respectively. No difference in active vitamin was found between the day of fabrication and after 30 days of storage, indicating that the gel was effective in limiting light and oxygen exposure. Moreover, the panelists showed a positive reception, with no unpleasant taste being noticed after the strawberry flavoring was added to the candies [59].

### 5.5. Consumer Acceptance and Sensory Properties of Plant-Based Emulsion Gels in Food

Consumer acceptance is one of the challenges hindering the mass adoption of plant-based emulsion gel in food. One major issue with plant-based products named by consumers lies with the heavy use of legume proteins, giving the product an undesirable “grassy” or “beany” odor and taste [129]. The addition of volatile additives enhanced consumer acceptance of plant-based emulsion gel. In spreadable plant-based cheese based on pea protein, inulin, and olive oil, the incorporation of essential oils leads to better odor perception in the sensory analysis [96]. In addition, fermentation may also reduce “beany“ perception to consumers [112,130]. Volatile analysis of fermented pea gel revealed that volatiles responsible for “grassy“ and “earthy“ odors are substantially reduced after inoculation and fermentation [130].

Despite the structural importance that polysaccharides provide to emulsion gels, the incorporation of polysaccharides may lead to adverse sensory properties. Insights may be drawn from primarily plant-based fat substitutes developed to improve the fatty acid profile in deli meats. Incorporating KGM emulsion gel into chorizo sausage led to a loss in the perception of juiciness and firmness, with an increasing degree at higher KGM content [131]. The lower perceived juiciness was attributed to KGM’s high WHC as less fluid was released from chewing. Similar results were seen across various plant-based polysaccharides and in mixed gels [50,132]. In a similar vein, oil release and mouthfeel of emulsion gel are also ingredient dependent. For instance, Hu et al. [133] demonstrated that at 5% oil content, the oiliness perception of modified starch/gellan gum gel was higher than that of whey protein/gellan gum gel at 20% oil content. The authors attribute this observation to the onset of enzymatic digestion of starch during mastication [133]. Therefore, consumer perception is shown to be formulation dependent on the types of biopolymers used and their respective ratios. To achieve desired product characteristics, plant-based emulsion gel requires extensive optimization.

## 6. Conclusions

The recent boost in the popularity of plant-based foods has been the primary driver in the development and understanding of plant-based emulsion gel as an ingredient or an entire food matrix. Plant-based ingredients are often perceived as healthier, more sustainable, and more environmentally friendly than food from animal sources. Plant proteins, polysaccharides, and lipids were shown in research to function synergistically in various configurations to deliver desirable qualities for a food product. As evidenced in this review, altering the types and amounts of protein, oil, and polysaccharide could change the emulsion gels’ rheology, texture, and thermal performance. Processing techniques, such as high-pressure homogenization and ultrasonication, have widened the scope for plant-based emulsion gel design. Essential qualities such as droplet size, stability, and bioavailability in functional emulsion gels could be substantially improved because of the choice of treatment. On the other hand, characterization techniques have allowed researchers to quantify and evaluate the properties of plant-based emulsion gel in detail. These techniques helped elucidate the properties of plant-based components (e.g., microscopy and DSC) and, critically, their applicability as a food analog (e.g., TPA).

Potential applications and possible future research were described in this study. Research has indicated that shortcomings in using plant-based ingredients may be improved using additives. Some food types, such as yogurt and mayonnaise, have been more successful in overcoming the challenges of using plant-based ingredients compared to other types of food. The development of functional foods using emulsion gel was also shown to be feasible in principle. Given the prevalence of emulsions and analogous structures in food, it is believed that creating a plant-based emulsion gel that could closely mimic the physical properties of their designated animal-based counterparts will be instrumental for the success of a plant-based alternative food product.

## Figures and Tables

**Table 1:** Techniques used for characterization of plant-based emulsion gel.

Properties	Techniques and Methodology		Characteristics of Emulsion Gel	Ref.
Appearance	Visual inspection	Color, gel fabrication, stability/instability	Color, gel fabrication, stability/instability	[89,90,91,92,93,94,95]
	Colorimetry	CIE-LAB color space coordinates	Quantitatively evaluate the color	[96]
		Image converter analysis software (ImageJ^®^)		[97]
Rheology	Small amplitude oscillatory shear (SAOS)	Frequency sweep	Viscoelasticity	[40,42,82,95,98,99,100]
		Strain sweep	Linear viscoelastic region (LVR)	
		Time sweep	Viscoelasticity evolution over time	
		Creep-recovery test	Transient viscoelastic behavior	
	Large amplitude oscillatory shear (LAOS)	Stain sweep Lissajous curve Fourier-transform rheology	Gel strength, viscoelastic behavior in the non-linear viscoelastic region	[92,95,101]
Texture	Texture profile analysis (TPA)		Hardness, springiness, gumminess, chewiness, cohesiveness, viscidity, and stiffness, gel strength	[40,42,82,92,93,97,99,102,103]
Microstructure	Droplet size distribution	Static Laser diffraction	Droplet size distribution	[91,100,104,105]
		Dynamic light scattering (DLS)		[82,92,98]
	Microscopy	Optical microscope (OM)	Droplet size and organization	[89,104,106,107]
		Polarized light microscope (PLM)	Structure of specific material (such as crystals and fibers)	[89,92,105]
		Confocal laser scanning microscope (CLSM)	Droplet size distribution, shape, and behavior	[75,93,97,98,99]
		Scanning electron microscope (SEM)	Structure of gel network	[40,95,98,102]
		Scanning electron cryo-microscopy (Cryo-SEM)	Structure of a well-maintained gel network	[95]
Stability	Thermal properties	Differential scanning calorimetry (DSC)	The amount of heat required to increase the temperature as a function of temperature	[75,82,92]
		Thermogravimetric analysis (TGA)	the change of the sample mass over time as the temperature changes	[42]
	Zeta potential		Stability	[82,100]
	Water holding capacity (WHC)	Moisture loss from centrifugation	Ability to retain water molecules	[42,75,82,98,100]
	Freeze-thaw cycling	Fluid loss from the freeze-thaw cycle	Stability under extreme temperature stress	[14,90,103]

**Table 2:** Summary of studies in food application of plant-based emulsion gel.

Target Food	Formulation	Summary	Ref.
Yogurt (Dairy)	Lentil protein isolate (LPI), sunflower oil, Yoflex^®^ Acidifix^TM,^ sucrose	Similar cohesiveness and viscosity but higher firmness and consistency than dairy yogurt. The fermented gel shows pale pink color. Low FODMAP content, suitable for irritable bowel syndrome.	[111]
	PPI, canola oil, GDL, Custom starter culture (MEGAN, VEGAN, ExECO)	VEGAN strain produced yogurt less associated with “cut herb” and “woody” and better associated with “coffee” and “smoked”. Reduction in volatiles associated with “grassy” odor.	[112]
	Potato protein isolate, sunflower oil, glucose, starter culture	High-pressure homogenization created highly stable emulsions at 1.5–10% oil. The whiteness of emulsion gel may be increased through higher pressure and oil content. Possible application in low-fat or Greek-style yogurt.	[76]
Cheese (Dairy)	Yellow pea/fava bean protein, canola oil, carrageenans (κ-, ι-) and xanthan gum, nutritional yeast, calcium sulfate	17.5% boiled fava bean flour and 1% κ- carrageenan were found to have indifferent springiness and chewiness to Gouda cheese but were also harder and less cohesive. Boiled yellow pea flour has the closest color resemblance to Gouda cheese.	[113]
	Zein, starches (corn, tapioca), sunflower oil	30% protein sample showed similar extensibility to the textural properties (hardness, chewiness, and gumminess) of cheddar cheese. Reduction in texture parameters at 50 °C is seen in both zein and cheddar cheese.	[114]
	Pea protein, pea fiber, potato fiber, sunflower oil/coconut fat, shea stearin, lactic acid, salt	Pea protein slurry with 15% emulsion gel showed a similar spreadability index to dairy cheese. Adding oregano and rosemary essential oils was able to reduce the perception of the grassy odor	[96]
Pork fat (Animal fat analog	SPI, fully hydrogenated canola oil, canola oil, transglutaminase oil	Softer than pork fat tissue. Thermal properties of animal fat may be achieved by blending solid plant fat with oil.	[115]
	SPI, KGM, coconut oil, transglutaminase	Similar hardness to pork fat (5% SPI, 4% KGM, and 10% oil (*w*/*w*)) Similar in color space to L* and b*	[50]
	DKG, MC, canola/coconut oil	MC and DKG complement each other in thermal properties at different temperatures. At 80 °C, coconut oil emulsion gel with a high MC better emulates the textural properties of pork fat.	[38]
Pork/beef fat (Animal fat analog)	Lecithin, potato starch (PS), inulin, soybean oil/coconut oil	At 12.8% (*w*/*w*) PS, soybean oil emulsion gel meltability is similar to that of pork fat. At the same PS content, coconut oil emulsion gel was similar to beef fat. Soybean/coconut oil emulsion gel with different PS contents may have a similar hardness to pork fat. Emulsion gels were consistently softer than beef fat.	[41]
Butter (Fat replacer)	Extra virgin olive oil, inulin, soy, lecithin	Harder product compared to control. Increasing emulsion gel content decreased spread during baking. Emulsion gel lacks plasticity and is therefore unable to create a porous structure.	[116]
Mayonnaise (Egg yolk)	Sunflower oil, chickpea protein/fava bean protein/yellow split lentils protein, xanthan gum, vinegar, sugar, salt, mustard powder	Xanthan gum is required to increase the viscosity and stability of the mayonnaise analog. Chickpea protein mayonnaise is indifferent to egg mayonnaise control. (Extrudability, compression texture analysis, color, and sensory analysis).	[117]
	Citrus fruit fiber, corn peptides, sunflower oil	HIPE has been characterized by above 90% thixotropic recovery. High heat and freeze-thaw stability are seen in HIPE. The creaminess and thickness of HIPE may be altered by fiber content.	[118]
Functional food	β-Carotene, zein, glycerol, and corn oil	β-Carotene loaded in the oil phase showed higher retention after UV treatment than in the aqueous phase. Additional β-carotene as an antioxidant in glycerol further increases retention in the oil phase.	[93]
	Rhamnogalacturonan-I enriched pectin, soybean oil, Tween 20, curcumin	Active filler pectin gel showed high thermal stability. The release of curcumin may be modified through gel structure, but no difference in bioavailability is seen.	[61]
Gummy candy (Functional foods)	Vitamin B_12_ and D_3,_ gum Arabic, inulin, flaxseed oil, pectin	No reduction in vitamin activity after 30 days of storage. No unpleasant taste was noted by panelists after the addition of the strawberry aroma.	[59]

## Data Availability

Not applicable.
